# Genistein Inhibits Fine-Dust-Induced Matrix Metalloproteinase-1 in Human Keratinocytes

**DOI:** 10.3390/ph18111750

**Published:** 2025-11-17

**Authors:** Dong Keun Song, Yun Young Jeong, Eunmiri Roh, Hyun Young Shin, Jong-Eun Kim

**Affiliations:** 1 Department of Food Science and Technology, Korea National University of Transportation, Jeungpyeong 27909, Republic of Korea; ehdrms0210@naver.com; 2 Convergence Program of Biohealth and Intelligence Technology, Department of IT·Energy Convergence, Graduate School, Korea National University of Transportation, Jeungpyeong 27909, Republic of Korea; yunyoungjeong727@a.ut.ac.kr; 3 Department of Cosmetic Science, Kwangju Women’s University, Gwangju 62396, Republic of Korea; roheun-miri@kwu.ac.kr; 4 Institute of Biotechnology and Bioindustry, Korea National University of Transportation, Jeungpyeong 27909, Republic of Korea; shydud0629@ut.ac.kr

**Keywords:** genistein, fine dust, matrix metalloproteinase-1, keratinocyte

## Abstract

**Background/Objectives:** Particulate matter (PM), which comprises airborne pollutants characterized by small sizes (typically from 5 to 8 μm in Korea), adversely affect skin health and accelerate aging by inducing oxidative stress and upregulating the expression of matrix metalloproteinase-1 (MMP-1), an enzyme responsible for collagen degradation. The skin, which is the largest organ and the primary barrier against harmful external stimuli such as air pollution, is particularly vulnerable to continuous PM exposure, which can cause skin aging and carcinogenesis. Given the effects of PM on skin aging, identifying compounds that can mitigate these adverse effects is crucial. Genistein is a naturally occurring isoflavone that has not been extensively studied in the context of PM-induced skin aging. **Methods:** In this study, we investigated the protective effects of genistein against PM-induced skin aging in HaCaT human keratinocytes. **Results:** Our results demonstrated that genistein treatment significantly reduced PM-induced MMP-1 expression, indicating a protective effect against collagen degradation. Additionally, genistein decreased the expression of the transcription factors activator protein-1 (AP-1) and nuclear factor kappa B (NF-κB), both of which are involved in the regulation of MMP-1. Furthermore, genistein markedly reduced the production of reactive oxygen species (ROS), a key marker of oxidative stress induced by PM exposure. **Conclusions:** These findings suggest that genistein exerts protective effects against PM-induced skin aging by attenuating collagen degradation and oxidative stress, indicating its potential as a therapeutic agent for improving skin aging associated with PM exposure.

## 1. Introduction

The skin, the body’s largest organ with a surface area of approximately 1.8 square meters, serves as a critical barrier against environmental pathogens and irritants [1]. Structurally, the skin consists of two primary layers, the epidermis and dermis, and a subcutaneous layer known as the hypodermis. The epidermis predominantly consists of keratinocytes arranged in four to five distinct layers, whereas the dermis contains nerves, blood vessels, glands, and hair follicles [2,3]. In particular, keratinocytes not only form a physical barrier but also play an essential role in maintaining skin homeostasis through immune regulation, wound healing, and the production of antimicrobial peptides [4]. This complex architecture, which encompasses these components, provides physical protection and plays a key role in immune defense and sensory perception.

Skin aging is a multifactorial process influenced by both endogenous and exogenous factors [5]. Endogenous factors such as genetics, age, and sex account for less than 20% of the aging process. In contrast, exogenous factors, which are collectively termed the “exposome,” contribute to approximately 80% of skin aging [6]. Exposomes encompass all environmental exposures and lifestyle factors, including ultra-violet radiation (UVR), air pollution, tobacco smoke, and nutrition [7]. Given the direct exposure of the skin to the external environment, it is particularly susceptible to aging processes driven by exogenous factors.

Airborne particulate matter (PM), which is collectively referred to as fine dust, has become a pressing environmental concern because of its profound influence on human health [8,9]. Although traditionally studied for its effects on the respiratory and cardiovascular systems, the effects of PM on the skin have received increasing attention [10]. Fine dust is a complex mixture of organic chemicals, heavy metals, and polycyclic aromatic hydrocarbons, all of which can interact with the skin either through direct deposition on the epidermal surface or by penetrating into the deeper layers [11]. These interactions initiate biological responses that accelerate the natural process of skin aging. One of the primary mechanisms linking fine dust exposure and skin aging is the generation of reactive oxygen species (ROS) [12,13]. Oxidative stress caused by ROS can damage cellular structures, impair DNA integrity, and disrupt lipid and protein functions in the skin barrier [13]. This process also stimulates the release of pro-inflammatory cytokines and activates signaling pathways that cause collagen degradation and reduced skin elasticity. Over time, these combined biological effects manifest as visible signs of skin aging, including wrinkles, hyperpigmentation, and dull skin appearance [14,15]. Moreover, chronic exposure to polluted environments exacerbates the cumulative burden of skin stressors. Fine dust can induce direct oxidative and inflammatory damage and also acts synergistically with UVR, amplifying photoaging processes [16]. This combined stress accelerates dermal matrix breakdown and disturbs skin repair [17]. With the growing urban populations and worsening air quality globally, the dermatological effects of fine dust have become an increasingly important area of public health research. Investigating these mechanisms is critical for developing preventive measures, designing new skincare strategies, and shaping environmental policies to reduce exposure.

Inhalation of PM is associated with a spectrum of adverse health effects, notably, cardiovascular diseases characterized by increased blood pressure, thrombosis, insulin resistance, vascular damage, and accelerated atherosclerosis [10,18]. However, in addition to its systemic effects, PM poses a direct risk to the skin. PM particles can penetrate the skin barrier through hair follicles and intercellular spaces, initiating oxidative stress through ROS production [12,19]. This oxidative stress triggers inflammatory responses, including the secretion of cytokines like tumor necrosis factor (TNF)-α, interleukin (IL)-1α, and IL-6, and upregulates matrix metalloproteinases (MMPs) such as MMP-1, MMP-2, and MMP-9 [20,21]. The resulting degradation of collagen and other components of the extracellular matrix (ECM) contributes to extrinsic skin aging and exacerbates dermatological conditions such as atopic dermatitis, acne, and psoriasis [11].

MMP-1 plays a pivotal role in the initial cleavage of interstitial collagen, which is fundamental to the structural integrity of the ECM [22,23]. Elevated MMP-1 activity, which is induced by environmental factors such as UVR and PM, disrupts the balance between collagen synthesis and degradation [24,25]. This imbalance leads to a net loss of collagen over time, which manifests as visible signs of skin aging such as wrinkles, sagging, and reduced elasticity [22,26]. Therefore, regulation of MMP-1 activity is essential for maintaining skin health and mitigating aging.

Genistein, a prominent isoflavone and phytoestrogen, exhibits a wide array of pharmacological properties [27,28]. This compound is predominantly found in soybeans and is commonly consumed through dietary sources [29,30]. Clinical evaluations involving adult women have also demonstrated its favorable safety profile after long-term administration [31]. From a molecular perspective, genistein functions as a tyrosine kinase inhibitor and exhibits antioxidant activity by scavenging free radicals and chelating metal ions [27,32]. The anti-inflammatory effects of genistein involve modulation of signaling pathways and suppression of pro-inflammatory cytokine production [33,34]. The anticancer potential of genistein has been explored in various human cell lines and animal models, where it has been shown to induce apoptosis, inhibit cell proliferation, and impede angiogenesis [34].

In dermatological research, genistein has shown promise in protecting against ultra-violet B (UVB)-induced skin damage [33]. Studies involving human keratinocytes (HaCaT cells) and murine models have revealed that genistein treatment can reduce UVB-induced inflammation and wrinkle formation following UVB exposure. In addition to its antioxidant and anti-inflammatory properties, genistein has been reported to enhance skin structure and repair through its interaction with the estrogen receptor β [35]. Collectively, these findings underscore the multifaceted protective roles of genistein in preserving skin integrity and promoting dermal homeostasis.

Despite these findings, however, the potential of genistein to counteract PM-induced skin aging remains unexplored. Since both UVR and PM exposure lead to increased ROS production and MMP activation, the antioxidant and anti-MMP properties of genistein can be plausibly considered to confer protective effects against PM-induced skin damage. Furthermore, the capacity of genistein to modulate inflammatory responses may mitigate the cytokine-mediated pathways involved in PM-induced skin aging.

In this study, we aimed to investigate the protective effects of genistein against PM-induced skin aging by using HaCaT cells as an in vitro model. We assessed the effects of genistein treatment on ROS generation, MMP-1 expression, and collagen degradation after PM exposure. By elucidating the mechanisms underlying the protective actions of genistein, we aimed to demonstrate its potential as a therapeutic agent for preventing or reducing the skin aging caused by environmental pollutants. This study could contribute to the development of novel skincare interventions and strategies aimed at enhancing skin resilience against the detrimental effects of air pollution.

## 2. Results

### 2.1. Effects of Genistein on PM-Induced MMP-1 Expression

**Figure 1:**
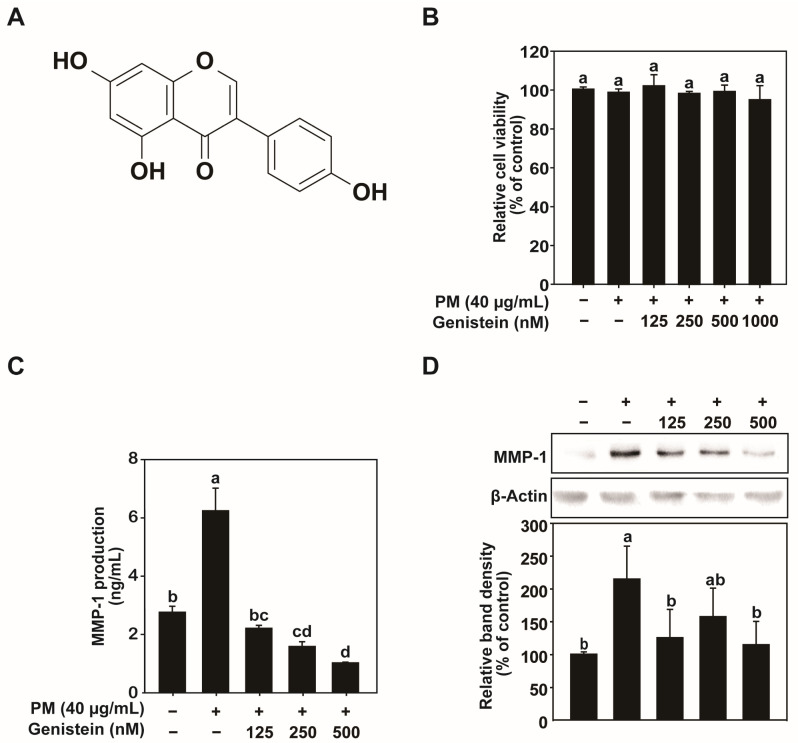
Effects of genistein on cell viability and MMP-1 expression in HaCaT cells. (**A**) Chemical structure of genistein. (**B**) Effect of genistein on HaCaT cell viability. HaCaT cells were treated with the indicated concentrations of genistein, and cell viability was assessed after 24 h using the MTT assay. (**C**,**D**) MMP-1 expression levels were evaluated by ELISA and western blot analyses. β-Actin was used as the loading control for MMP-1 expression. Data are expressed as the mean ± standard deviation (SD) from three independent biological experiments. Different letters (a–d) indicate statistically significant differences among groups as determined by one-way analysis of variance (ANOVA) followed by Duncan’s multiple range test at *p* < 0.05. The PM/Genistein—group represents the DMSO vehicle control.

Before evaluating the protective effects of genistein against PM-induced skin aging, we assessed its cytotoxicity and effects on MMP-1 expression in HaCaT cells. Assessing cytotoxicity was important to ensure that any reduction in extracellular markers such as MMP-1 reflected genuine biological effects rather than nonspecific changes caused by abnormal cellular responses. The cells were treated with genistein (Figure 1A) at concentrations of 125, 250, 500, or 1000 nM in the presence of PM (40 μg/mL). After 24 h of treatment, the cell viability remained above 80% at all genistein concentrations (Figure 1B). Although a slight decrease in viability was observed at 1000 nM, this change did not indicate cytotoxicity. However, because the biological response at this concentration could not be clearly interpreted, concentrations of 125, 250, and 500 nM were used for subsequent experiments to maintain reliable biological relevance.

**Figure 2:**
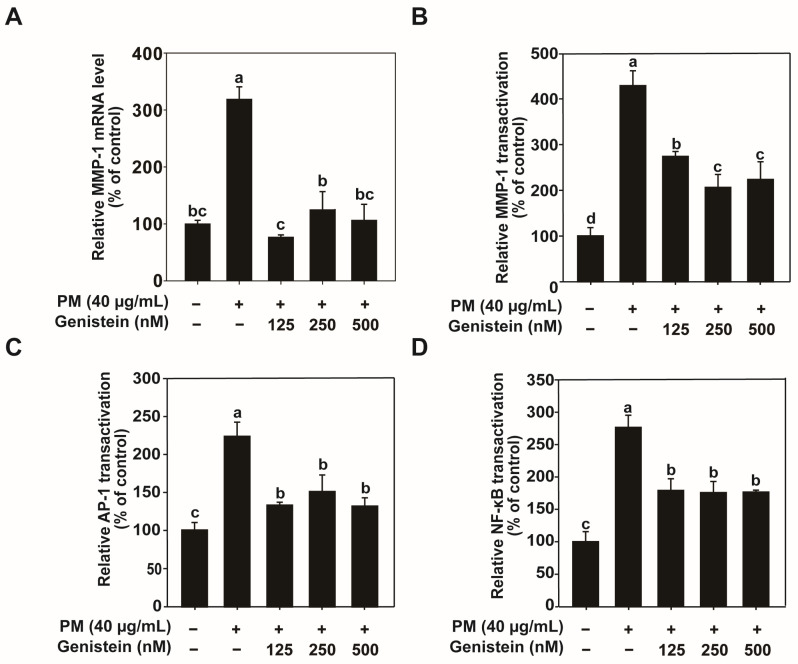
Genistein attenuated PM-induced MMP-1 mRNA expression and MMP-1 promoter activity by suppressing AP-1 and NF-κB transactivation in HaCaT cells. (**A**) MMP-1 mRNA expression was quantified by qRT-PCR. (**B**–**D**) MMP-1, AP-1, and NF-κB promoter activities were evaluated using GFP reporter assays. Data are expressed as the mean ± standard deviation (SD) from three independent biological experiments. Different letters (a–d) indicate statistically significant differences among groups as determined by one-way analysis of variance (ANOVA) followed by Duncan’s multiple range test at *p* < 0.05. The PM/Genistein—group represents the DMSO vehicle control.

PM exposure markedly elevated MMP-1 protein and mRNA expression in HaCaT cells. Genistein treatment effectively reduced the PM-induced increase in MMP-1 expression in a dose-dependent manner, as demonstrated by enzyme-linked immunosorbent assay (ELISA), western blot, and quantitative polymerase chain reaction (qPCR) analyses (Figure 1C,D and Figure 2A). To further elucidate the mechanisms underlying these findings, we assessed MMP-1 promoter activity using a green fluorescent protein (GFP) reporter gene assay. The results showed that PM significantly enhanced the MMP-1 promoter activity, which was attenuated by genistein treatment (Figure 2B). These findings indicate that genistein suppresses PM-induced MMP-1 expression by inhibiting the transcriptional activation of the *MMP-1* gene.

### 2.2. Genistein Modulates PM-Induced Activation of Activator Protein-1 and Nuclear Factor Kappa B

Activator protein-1 (AP-1) and nuclear factor kappa B (NF-κB) are key transcription factors involved in the regulation of MMP-1 expression [8]. We investigated how genistein regulates the activation of transcription factors in response to PM exposure. PM treatment significantly increased the promoter activities of AP-1 and NF-κB in HaCaT cells. Genistein treatment effectively suppressed the PM-induced activation of both AP-1 and NF-κB promoters (Figure 2C,D). These results suggest that genistein downregulates MMP-1 expression by inhibiting the activation of the transcription factors AP-1 and NF-κB.

### 2.3. Genistein Inhibits PM-Induced Activation of the Mitogen-Activated Protein Kinase Pathway

**Figure 3:**
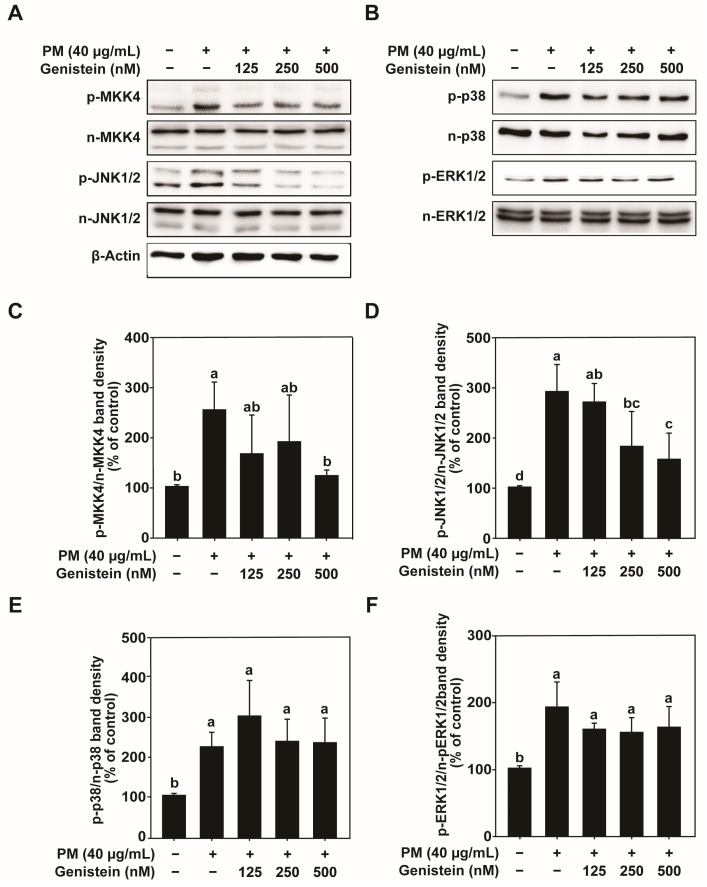
Inhibitory effects of genistein on PM-induced signaling pathways in HaCaT cells. (**A**) Effect of genistein on the phosphorylation of MKK4 and JNK1/2, as determined by western blotting. (**B**) The PM-induced phosphorylation of p38 and ERK was analyzed by western blotting. (**C**–**F**) Protein levels of (**C**) p-MKK4, (**D**) p-JNK1/2, (**E**) p-p38, and (**F**) p-ERK were quantified. The expression levels of phosphorylated proteins were quantified relative to their corresponding total forms. Data are expressed as the mean ± standard deviation (SD) from three independent biological experiments. Different letters (a–d) indicate statistically significant differences among groups as determined by one-way analysis of variance (ANOVA) followed by Duncan’s multiple range test at *p* < 0.05. The PM/Genistein—group represents the DMSO vehicle control.

The mitogen-activated protein kinase (MAPK) signaling pathway plays a crucial role in mediating cellular responses to environmental stress, including the regulation of AP-1 and NF-κB activity [36,37]. We investigated the phosphorylation of MAPK family members to clarify the molecular mechanisms underlying the protective effects of genistein in HaCaT cells exposed to PM. PM exposure led to increased phosphorylation of mitogen-activated protein kinase 4 (MKK4) and c-Jun N-terminal kinase 1/2 (JNK1/2), indicating activation of the JNK pathway (Figure 3A,C,D). Pretreatment with genistein significantly reduced the PM-induced phosphorylation of MKK4 and JNK1/2.

In contrast, genistein treatment did not significantly alter the phosphorylation of p38 MAPK and extracellular signal-regulated kinase (ERK) (Figure 3B,E,F). These findings suggest that genistein selectively inhibits PM-induced activation of the MKK4-JNK1/2 axis within the MAPK pathway, thereby suppressing the expression of downstream transcription factors and MMP-1.

### 2.4. Genistein Alleviates PM-Induced Oxidative Stress

**Figure 4:**
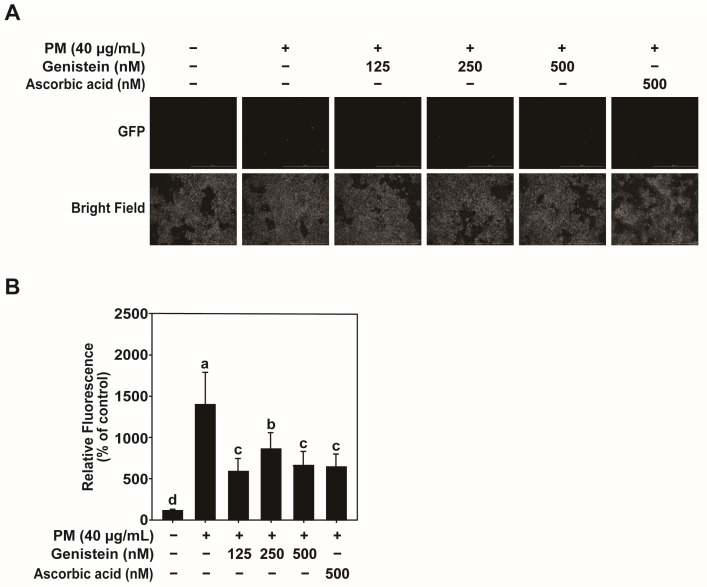
Genistein reduced the PM-induced oxidative damage in HaCaT cells. (**A**) Images of the effects of PM-induced ROS reduction by genistein imaged by Cytation 5 using the DCF-DA assay (scale bar = 1000 µm). (**B**) Quantification of intracellular ROS levels in PM-induced HaCaT cells. Data are expressed as the mean ± standard deviation (SD) from three independent biological experiments. Different letters (a–d) indicate statistically significant differences among groups as determined by one-way analysis of variance (ANOVA) followed by Duncan’s multiple range test at *p* < 0.05. The PM, Genistein, and Ascorbic acid—groups represent the DMSO vehicle control. Ascorbic acid was used as a positive control. GFP, green fluorescent protein.

Oxidative stress plays a pivotal role in PM-induced skin aging by promoting the production of ROS, which can damage cellular components and activate signaling pathways, leading to collagen degradation [20]. We used the DCF-DA assay to measure intracellular ROS levels in HaCaT cells exposed to PM. The ROS production in the PM-treated cells was significantly greater than that in untreated controls. Genistein pretreatment markedly reduced the ROS levels induced by PM exposure (Figure 4A,B). These findings suggest that genistein exerts antioxidant effects, thereby protecting HaCaT cells from PM-induced oxidative damage and subsequent skin aging. A schematic overview of the proposed mechanism underlying these effects is provided in Supplementary Figure S1.

## 3. Discussion

Environmental pollution, especially air pollution driven by rapid urbanization and industrialization, is an escalating global health concern [14,38]. PM, a complex mixture of solid and liquid particles suspended in air, is one of the most harmful components of air pollution [37]. The ability of PM to penetrate the skin barrier and induce oxidative stress and inflammation is critical for extrinsic skin aging [12]. This study aimed to explore the protective effects of genistein, a naturally occurring iso-flavone commonly found in soy products, against PM-induced skin damage by using HaCaT cells as an in vitro model.

Our findings confirmed that exposure to PM significantly elevated oxidative stress in HaCaT cells, which was indicated by the increased production of ROS. Excessive ROS is a well-documented catalyst of cellular damage, triggering pathways that disrupt collagen synthesis and promote the expression of MMPs, particularly MMP-1 [15]. MMP-1 plays a key role in breaking down collagen, a structural protein critical for maintaining the integrity of the skin, leading to visible signs of aging such as wrinkles and loss of elasticity [24,25]. These results are consistent with those of previous studies linking PM exposure to skin aging through oxidative stress and collagen degradation.

Importantly, our findings demonstrated that genistein effectively attenuates the detrimental effects of PM exposure by significantly reducing ROS levels. These results highlight the potent antioxidant activity of genistein. By scavenging ROS, genistein prevents the activation of downstream signaling pathways that lead to increased MMP-1 expression and subsequent collagen breakdown. This is crucial for preserving skin structure and delaying the onset of aging symptoms induced by environmental pollutants.

In addition to its antioxidant effects, genistein also modulates inflammatory pathways. Our results showed that genistein inhibited the activation of transcription factors such as AP-1 and NF-κB, both of which play pivotal roles in regulating inflammation and MMP-1 expression [39]. The suppression of these transcription factors by genistein leads to a reduction in the levels of pro-inflammatory cytokines, including TNF-α, IL-1α, and IL-6 [36]. By mitigating the inflammatory response, genistein not only protects the skin from damage, but also helps maintain homeostasis in an environment rich in pollutants.

The broad impact of environmental pollutants such as PM on skin health extends beyond premature aging, since PM exposure also exacerbates skin disorders such as atopic dermatitis, acne, and psoriasis [11]. Our findings showing the protective effects of genistein indicate its potential as a therapeutic agent that can be incorporated into skincare products designed to shield the skin from environmental stressors. This is particularly relevant for individuals living in urban settings with high pollution levels, where the skin is constantly exposed to harmful air-borne particles.

The protective effects of genistein may be attributed to its polyphenolic structure, which confers potent antioxidant and anti-inflammatory properties. By neutralizing free radicals, chelating metal ions involved in ROS generation, and suppressing the phosphorylation of MAPK signaling components (MKK4–JNK1/2), genistein mitigates oxidative stress–mediated pathways that induce MMP-1 expression and collagen degradation [22,40], thereby helping to preserve skin integrity under PM exposure. This regulatory capacity underscores the multifaceted mechanisms through which genistein protects the skin from environmental stressors. While the present study primarily focused on MMP-1 regulation, elastase also plays a role in skin aging and will be considered in future investigations.

Although previous studies have extensively explored the protective role of genistein against UV-induced skin damage [33], its potential to counteract PM-induced aging remains underexplored. Our research fills this gap by confirming that genistein reduces the oxidative stress caused by PM exposure and prevents the degradation of collagen, a critical component in maintaining skin elasticity and firmness.

Given the rising levels of pollution worldwide, particularly in rapidly urbanizing regions, this study has major implications. The incorporation of genistein into topical skin-care formulations offers a promising strategy for protecting the skin from the adverse effects of environmental pollutants. Future studies should focus on in vivo assessments and clinical trials to characterize the long-term efficacy and safety of genistein in humans. Considering its metabolism and bioavailability in physiological systems, such investigations will be essential to validate its therapeutic relevance and ensure safe translational application. Moreover, optimizing delivery systems to enhance skin penetration could further improve the protective effects of genistein. Future mechanistic studies are also required to determine whether genistein directly inhibits upstream kinases within the MKK4–JNK1/2 axis or primarily exerts its effects indirectly through oxidative stress attenuation. Nevertheless, topical formulations enriched with genistein, particularly in combination with other antioxidants or anti-inflammatory compounds, may offer a promising strategy for comprehensive protection against pollution-induced skin aging.

## 4. Materials and Methods

### 4.1. Chemicals and Reagents

High-glucose Dulbecco’s Modified Eagle Medium (DMEM; LM 001-05), penicillin–streptomycin solution (LS 202-02), and trypsin-ethylenediaminetetraacetic acid (EDTA) solution (LS015-02) were obtained from Welgene (Gyeongsan, Republic of Korea). Fetal bovine serum (FBS; FP-0500-A) was purchased from Atlas Biologicals (Fort Collins, CO, USA). Genistein (≥98% purity) was purchased from ChemCruz (Santa Cruz, CA, USA; sc-3515). Standardized particulate matter (PM_10-like; European Reference Material ERM-CZ100) was obtained from the European Commission Joint Research Centre (Brussels, Belgium). MMP-1 antibody was purchased from R&D Systems Inc. (Minneapolis, MN, USA). Antibodies against total ERK 1/2, total p38 MAPK, and total c-Jun N-terminal kinase 1/2 (JNK1/2) were obtained from Santa Cruz Biotechnology (Santa Cruz, CA, USA). Additional antibodies were purchased from Cell Signaling Technology (Danvers, MA, USA). Packaging vectors (psPAX2 and pMD2.G) and reporter plasmids, including pGF-AP-1-mCMV-EF1-Puro, pGF-NF-κB-mCMV-EF1-Puro, and pGF-MMP-1-mCMV-EF1-Puro (System Biosciences, Palo Alto, CA, USA), were acquired from Addgene Inc. (Cambridge, MA, USA). Enhanced chemiluminescence (ECL) Prime Western Blotting Detection Reagent was purchased from Amersham (Little Chalfont, UK). All the other chemicals and reagents were of analytical grade.

### 4.2. Cell Culture

HaCaT human keratinocytes were obtained from CLS Cell Lines Service GmbH (Heidelberg, Germany). The cells were cultured in DMEM supplemented with 10% FBS and 1% penicillin–streptomycin at 37 °C in a humidified atmosphere containing 5% CO_2_. HaCaT cells were used between passages 5–20 and were routinely tested and confirmed negative for mycoplasma contamination on a regular basis.

### 4.3. Preparation of PM

The PM reference material (mean aerodynamic diameter < 10 µm) was suspended in dimethyl sulfoxide (DMSO) to prepare a 25 mg/mL stock solution. The suspension was then sonicated for 15 min to prevent particle agglomeration. Before use, the PM stock was diluted in serum-free DMEM to the desired concentrations for experimental treatments, with a final concentration of 40 μg/mL used in all assays.

### 4.4. Cell Viability Assay

Cell viability was assessed using the 3-(4,5-dimethylthiazol-2-yl)-2,5-diphenyltetrazolium bromide (MTT) assay (Thermo Fisher Scientific, Waltham, MA, USA; M6494). HaCaT cells were seeded at a density of 1.5 × 10^5^ cells/mL in 96-well plates and cultured in DMEM containing 10% FBS and 1% penicillin–streptomycin for 24 h at 37 °C with 5% CO_2_. Upon reaching approximately 80% confluence, the cells were washed twice and incubated in serum-free DMEM for an additional 24 h under the same conditions. The cells were treated with various concentrations of PM and genistein (125, 250, 500, or 1000 nM) in serum-free DMEM for 24 h. After treatment, 10 μL of MTT solution (0.45 mg/mL) was added to each well, and the plates were incubated for 2 h at 37 °C. The supernatant was carefully removed, and 100 μL of DMSO was added to dissolve the formazan crystals. The absorbance was measured at 570 nm using a microplate reader (Biotek Instruments, Winooski, VT, USA) to determine cell viability. Genistein stock solutions were prepared in DMSO and diluted with serum-free DMEM prior to treatment. The same concentration of the DMSO vehicle was used as the control group.

### 4.5. Enzyme-Linked Immunosorbent Assay

Before the MTT assay, culture supernatants were collected for ELISA analysis. The concentration of MMP-1 in the culture supernatants was quantified using ELISA kits (DY901B) according to the manufacturer’s instructions. Absorbance was measured at 450 nm with correction at 540 nm using a microplate reader (Biotek Instruments).

### 4.6. Western Blot Analysis

HaCaT cells were seeded at a density of 2 × 10^5^ cells/mL in 6-well plates and cultured as described above. After serum starvation for 24 h, the cells were pretreated with genistein (125, 250, or 500 nM) in serum-free DMEM for 1 h, followed by exposure to PM for 24 h. The cells were then washed twice with cold phosphate-buffered saline (PBS) and lysed in ice-cold lysis buffer (Cell Signaling Technology; 9803) containing 50 mM Tris-HCl (pH 8.0), 150 mM NaCl, 1% NP-40, 0.1% sodium dodecyl sulfate (SDS), 0.5% sodium deoxycholate, 1 mM dithiothreitol, 1 mM phenylmethylsulfonyl fluoride, and 1 mM sodium orthovanadate. Cell lysates were collected by scraping and centrifuged at 13,000 rpm for 10 min at 4 °C, and the supernatants were collected. Protein concentrations were determined using a DC Protein Assay Kit (Bio-Rad Laboratories, Hercules, CA, USA; 5000002). Equal amounts of protein were separated by 10% SDS-polyacrylamide gel electrophoresis and transferred onto 0.2 μm polyvinylidene difluoride (PVDF) membranes (Amersham; 10600021). Membranes were blocked with 5% non-fat milk in Tris-buffered saline containing 0.1% Tween-20 (TBS-T) for 1 h at room temperature, and then incubated overnight at 4 °C with primary antibodies. After washing three times with TBS-T, the membranes were incubated with horseradish peroxidase (HRP)-conjugated secondary antibodies (GenDEPOT; Barker, TX, USA) diluted in 5% non-fat milk for 1 h at room temperature. Protein bands were visualized using ECL Prime Western Blotting Detection Reagent (Amersham) and analyzed using the FUSION Solo S imaging system (Vilber Lourmat, Collégien, France). After primary detection, membranes were stripped and reprobed for the loading control. Band intensities were quantified within the linear range using the imaging system’s built-in analysis module and were subsequently normalized to the corresponding loading control or total protein signals. Detailed information on the specific antibodies is provided in Table S1.

### 4.7. Quantitative Real-Time PCR

HaCaT cells were seeded at a density of 2 × 10^5^ cells/mL in 6-well plates and cultured as described above. After serum starvation for 24 h, the cells were pretreated with genistein (125, 250, or 500 nM) in serum-free DMEM for 1 h, followed by exposure to PM for 24 h. The cells were then washed twice with cold PBS, and total RNA was extracted. The RNA concentration and purity were subsequently assessed using a Take3 Micro-Volume Plate and a microplate reader (BioTek Instruments). First-strand complementary DNA (cDNA) was synthesized from total RNA using the ReverTra Ace™ qPCR RT Master Mix (Toyobo, Osaka, Japan; FSQ-201) in accordance with the manufacturer’s protocol. Quantitative real-time PCR (qRT-PCR) was performed using SYBR Green Real-Time PCR Master Mix (Toyobo; QPK-201) and gene-specific primers for MMP-1, with relative expression levels normalized to those of GAPDH. The reference gene showed stable Ct values across all treatment groups, supporting its appropriateness for data normalization. The specific primer sequences used for qRT-PCR were as follows: MMP-1 forward, 5′-CCC CAA AAG CGT GTG ACA GTA-3′; MMP-1 reverse, 5′-GGT AGA AGG GAT TTG TGC G-3′; GAPDH forward, 5′-GAG TCA ACG GAT TTG GTC GT-3′; and GAPDH reverse, 5′-TTG ATT TTG GAG GGA TCT CG-3′. Primer specificity was confirmed by melt-curve analysis showing a single peak, and primers were designed to span exon–exon junctions to avoid genomic DNA amplification.

### 4.8. GFP Reporter Gene Assay

HEK293T cells were co-transfected with the packaging vectors psPAX2 and pMD2.G, and the reporter plasmids pGF-AP-1-mCMV-EF1-Puro, pGF-NF-κB-mCMV-EF1-Puro, or pGF-MMP-1-mCMV-EF1-Puro using jetPEI transfection reagent (Polyplus Transfection, Illkirch, France) according to the manufacturer’s instructions. After 24 h, the transfection medium was replaced with fresh medium, and the cells were incubated for an additional 24 h. Viral particles were harvested by filtering the culture supernatant through a 0.45-μm syringe filter. HaCaT cells were infected with the viral particles in the presence of 8 μg/mL polybrene (EMD Millipore, Billerica, MA, USA) and incubated overnight. To eliminate non-transduced cells and ensure consistent infection across groups, cells were selected with 2 μg/mL puromycin (Sigma-Aldrich, St. Louis, MO, USA) for 24 h, resulting in only transduced cells remaining for analysis. Cells were treated with various concentrations of PM and genistein (125, 250, or 500 nM) in serum-free DMEM for 24 h. GFP fluorescence was measured using a Cytation 5 imaging system (Biotek Instruments) at excitation and emission wavelengths of 469 and 525 nm, respectively. Fluorescence values were background-corrected by the instrument’s built-in software and subsequently normalized to cell number.

### 4.9. Measurement of ROS

Intracellular ROS levels were measured using the DCF-DA (Thermo Fisher Scientific; D399) assay. HaCaT cells were seeded at a density of 1.5 × 10^5^ cells/mL in black 96-well plates and cultured as described above. After serum starvation for 24 h, the cells were incubated with 100 μM DCF-DA in Hank’s Balanced Salt Solution (HBSS) for 30 min at 37 °C. Excess DCF-DA was removed by washing the cells twice with HBSS. Cells were then treated with various concentrations of PM and genistein (125, 250, and 500 nM) diluted in HBSS and incubated for 45 min at 37 °C. Fluorescence intensity, which was indicative of ROS levels, was measured using a micro-plate reader (Cytation 5) at excitation and emission wavelengths of 469 and 525 nm, respectively. Ascorbic acid (500 nM, Sigma-Aldrich; A4544) was used as a positive control to validate the ROS detection system. Background-corrected fluorescence values were normalized to cell number using the instrument’s built-in analysis software, ensuring accurate ROS quantification independent of cell density.

### 4.10. Statistical Analysis

All experiments were performed in three independent biological replicates, and data are presented as the mean ± standard deviation (SD). Statistical analyses were conducted using SPSS Statistics software (version 21.0; IBM Corp., Armonk, NY, USA). Differences among groups were analyzed using one-way analysis of variance (ANOVA), followed by Duncan’s multiple-range test for post-hoc comparisons. The data were assumed to meet the normality and homogeneity-of-variance assumptions underlying ANOVA. Statistical significance was set at *p* < 0.05.

## 5. Conclusions

This study provides compelling evidence that genistein effectively combats PM-induced skin aging by reducing oxidative stress and preventing ECM degradation. These findings highlight the potential of genistein as a promising candidate for interventions to mitigate the harmful effects of environmental pollutants on skin health. As air pollution levels continue to rise, the utilization of natural compounds such as genistein offers a valuable and practical approach to safeguard skin integrity and promote overall skin health.

## Data Availability

Dataset available on request from the authors. The data are not publicly accessible due to internal data-management limitations.

## Supplementary Materials

The following supporting information can be downloaded at: https://www.mdpi.com/article/10.3390/ph18111750/s1, Figure S1: Proposed mechanism by which genistein attenuates PM-induced MMP-1 expression in HaCaT keratinocytes; Table S1: List of primary antibodies used for Western blot analysis.
