# Supplementary material for: Genistein Inhibits Fine-Dust-Induced Matrix Metalloproteinase-1 in Human Keratinocytes
Source: Pharmaceuticals (Basel). 2025 Nov 17;18(11):1750. doi: 10.3390/ph18111750 (PMC12655756; doi:10.3390/ph18111750)
Supplement: Supplementary file 1 [file pharmaceuticals-18-01750-s001.zip › pharmaceuticals-3949926-supplementary.pdf]

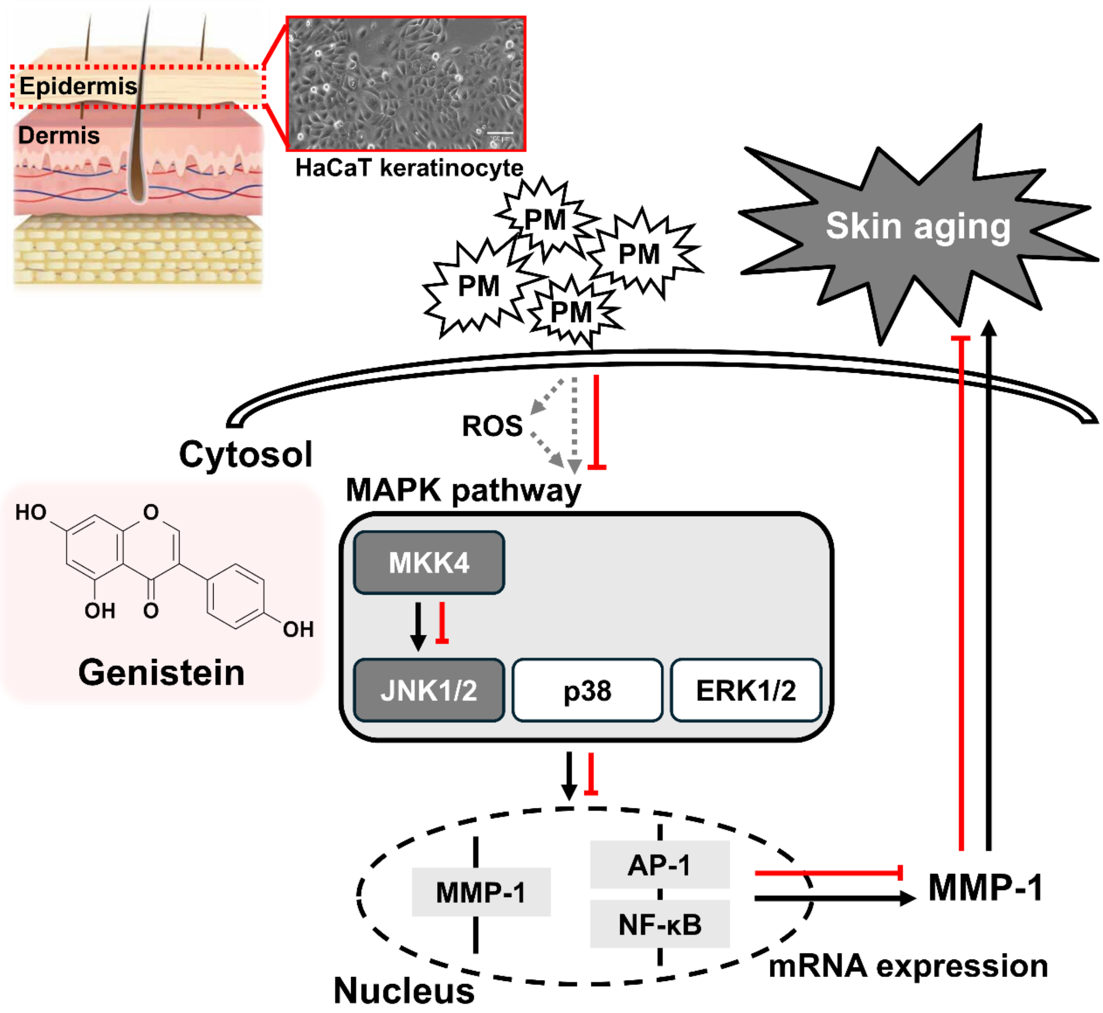

Figure S1. Proposed mechanism by which genistein attenuates PM-induced MMP-1 expression in HaCaT keratinocytes.

Table S1. List of primary antibodies used for Western blot analysis.

**Specific antibody**	**Catalog number**	**Host species**	**Dilution ratio**	**Company**
Matrix metalloproteinase-1	MAB901	Mouse		R&D Systems Inc. (Minneapolis, MN, USA)
β-actin	sc-47778	Mouse		Santa Cruz Biotechnology (Santa Cruz, CA, USA)
Phospho-SEK1/MKK4	9156	Rabbit		
SEK1/MKK4	9152	Rabbit		
Phospho-SAPK/JNK	9251	Rabbit	1:1000	Cell Signaling Technology (Danvers, MA, USA)
SAPK/JNK	9252	Rabbit		
Phospho-p38 MAPK	9211	Rabbit		
p38 MAPK	sc-7972	Mouse		Santa Cruz Biotechnology
Phospho-p44/42 MAPK	9101	Rabbit		Cell Signaling Technology
p44/42 MAPK	sc-514302	Mouse		Santa Cruz Biotechnology
Goat anti-Rabbit IgG(H+L)-HRP	SA002-500	Goat	1:5000	GenDEPOT (Barker, TX, USA)
Goat anti-Mouse IgG(H+L)-HRP	SA001-500	Goat		
